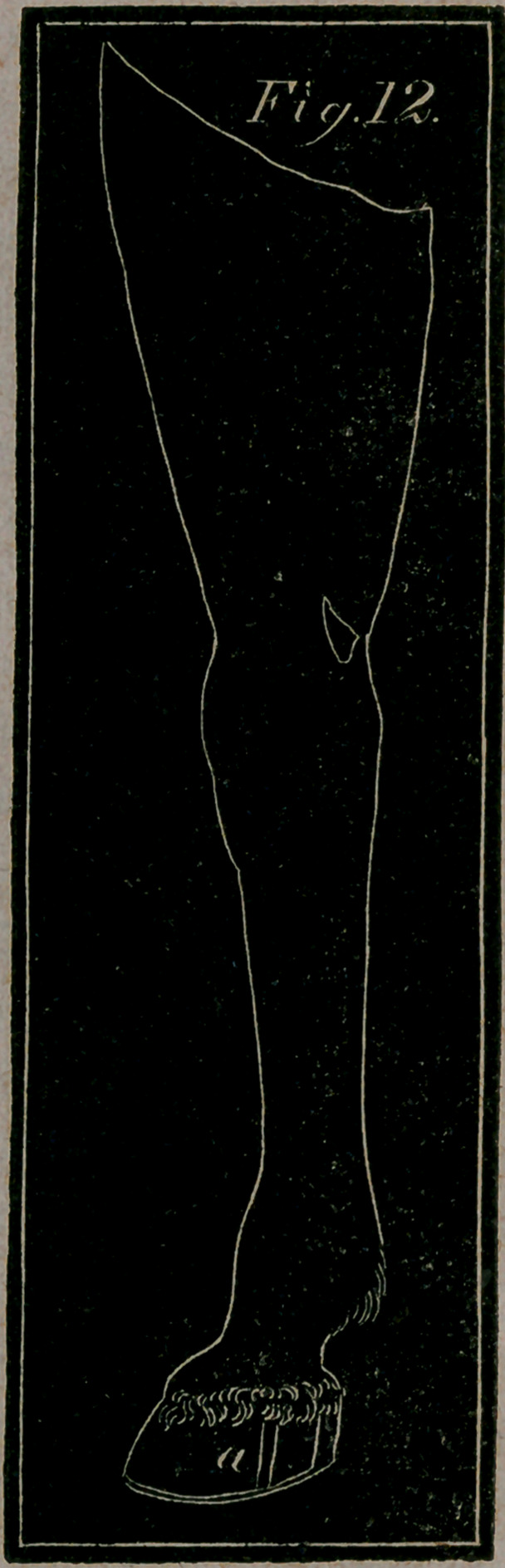# A Treatise on the Veterinary Pathology of the Different Lamenesses to Which the Horse Is Subject

**Published:** 1818

**Authors:** James Carver


### A
### TREATISE
### ON THE
### VETERINARY PATHOLOGY
### OF THE
### DIFFERENT LAMENESSES
### TO WHICH THE HORSE IS SUBJECT.

### INTRODUCTION.

OF all the quadruped animals, the Horse seems not only the most beautiful, but the most useful to the service of man; and, as no labour can be done, or business carried on, but by the assistance of this noble animal, he certainly claims our attention, to relieve him from the different lamenesses and infirmities to which we expose him, by reducing him from a state of nature to a life of art; for a horse in town, and a horse in the country, is, in many respects, no longer the same animal. These circumstances considered, we cannot pass over, without some attention, the merit his services deserve. We shall endeavour, therefore, to proceed to the cause, progress, treatment and cure of his diseases: such as *Corns—Quittors—Sand-cracks—Thrush—Grease—Spavins—Splints—Curbs—Ring-bones—Sprains,* and *Wind-galls;* to which the legs and feet of horses, from bad shoeing and mismanagement are so subject.

Lameness, signifies any disease or complaint of an animal, by which a free progression of his movements are impeded.

The steps of a horse in a sound state are equal and uniform, constituting a certain harmony subsisting in the movements of his whole body and limbs; any deviation therefore from this harmony, or defect in the moving his legs, constitutes lameness. Various are the causes which may occasion this, and frequently a combination of causes may take place at one and the same time, in different parts of the same limb; for instance, a prick from a nail in the foot, a strain in the tendons of the leg, the ligaments of the joints, or in the shoulders, frequently happen together; for as the pain arising from the prick, in the sensible lameness of the foot, causes the horse to trip and stumble, an exertion to save the pained foot may, and frequently does, occasion the straining of the ligaments of the joints or tendons, &c. of the legs; and thereby a combination of causes of lameness are produced.

The same effects are likewise produced from bony excrescences on the legs, occasional pain, or even an exertion of the animal to save the pained limb in such a case, may occasion the straining of some of the muscles in the shoulders; though lameness in this part seldom or ever happens to this bone, from the nature of its strength of structure and junction with the ribs; for the scapula is not fixed to the body, like many other parts, by joints, but by apposition; that is, laid along the outside of the ribs, and there fastened to, by the muscles, &c. which lie both on inside and outside of the shoulder blade: hence, though the muscles and tendons of the shoulder may sometimes be overstretched or strained, the bone can never be slipped out (as many, from want of a knowledge of its structure, often think it is) without force sufficient to destroy the texture of the parts which connect the latter with the ribs. It is true, that the ligaments which surround the articulation where the humerus joins with the scapula, at the *point of the shoulder*, is exposed to considerable injury from strains, &c. as is the case with other joints; and I have repeatedly heard professor Coleman say, as well as Mr. Bracy Clark, that they never knew or heard of a single instance of its being dislocated in a horse, though it must be allowed that the thing is possible: hence therefore it is, that not one lameness of a thousand, which, by the ignorant is pronounced lame in the shoulder, but that it is in the foot or feet, and not in that part.

Lameness also frequently proceeds from tumours or enlargements growing upon the bones of the legs of horses. These are commonly termed splints—spavins—wind-galls—ring-bones, &c. as is seen and represented in the different plates of this work, and which are distinguished or named from the *particular part*

*on which they grow.* But as these bony excrescences, or exostoses, as they are called in human pathology, are not limited to any part of the leg, as in some cases only, and are likely to grow on other bones, they are sometimes concealed in some situations impossible, except by dissection, to be discovered.

It sometimes also happens, that the exostoses or enlargements take place on the small bones of the joints, especially on those of the hock, the osseous matter forming a number of small tumours between the joints, and thereby cement two or more of those bones together, which, by impeding the action of the joint, causes permanent lameness.*

Many persons think and flatter themselves that they can cure these bony excrescences, by blistering, &c. even after they have acquired their utmost solidity. But those who are better acquainted with anatomy and structure of parts, will never make any

* This was the case with Old Messenger, who threw out a ring-bone when a colt of two years old, and which was the occasion of his coming to this country. This never being properly attended to, it became ossified, and produced ring-bone or ancalloses of the joint. This circumstance cannot be doubted, having the *bone now in my own possession*, which was dug up on Cock farm, on Long island, last summer, where *he died, in the presence of Mr. Cock and others*, some years ago.

such absurd assertions. I cannot forbear in this place to remark, that it is positively asserted that a celebrated self-dubbed veterinarian of the present day, and now in this city, does positively assert that he performs a cure of this disease by chiselling off the part, or rather the bony enlargement, from the main bone. How weak must that man be, who will so foolishly expose his ignorance of anatomy by such an assertion, being a flat contradiction to the nature of things. It is true, that in some cases, where the tumour has a very narrow base, it may be taken off with a chisel; but it must in all cases where it is done, not only be attended with serious and dangerous consequences, but much more detriment and injury to the horse, than the excrescence would be, if left in its fullest growth: indeed a caries of the bone must take place, and the cicatrix that remains must disfigure the horse more than the bony excrescence would have done in its fullest extent. Splints of uncommon size we daily see on horses' legs, yet they do not go lame; hence, therefore, it is obvious it is not the size of a splint that causes lameness, after it has grown to the full extent, but rather its interference with other parts that are subservient to the motion of the legs. As I have attacked the professional talents of a man, who, having assumed to a knowledge of a profession he cannot, from the very nature of his education, have a proper knowledge of what he aspires to, (a Veterinary Surgeon and Professor of Animal Medicines,) I deem it my duty to advocate the cause of truth, as it may be the means of saving some poor animal from his hands. Having made this bold assertion, and that before a body of scientific men, whose talents and knowledge of anatomy of parts are too well known and established to need any encomium of mine, I deem it therefore my duty to explain the nature of this chisel operation, and the impossibility of its being performed without injury to the animal. Caries, therefore, of a bone, is meant to *abrase*, *dig*, or *cut* one bony excrescence from another; the consequence of which is nothing more than a partial mortification of the bone, which separates the bond of union from the sound part, sooner or later. Dr. Hunter, Dr. Munro, Dr. Cullen and Dr. Coleman term every species of caries attended with loss of substance an *ulcer;* and this corrupted state of the bone happens when it has lost its living principle, which must be the case in the loss of the periosteum, from which cause, having lost that supply of natural colour, it becomes yellow, brown, and at last, when approaching to mortification, black. This, when the bone is corroded, causes a discharge of sanies which consumes the adjacent flesh, and by causing inflammation of the surrounding periosteum, causes great pain to the animal. I now wish it to be understood, that the attack I here make is to expose error; not upon the person, but upon the erroneous opinion and practice; for we should war against error in our brother as against our enemy, when it is the cause of truth we seek, and no personal motive influences us.

It likewise frequently happens, that these excrescences grow between or upon the large and small metacarpal, or cannon bone of the fore legs, and sometimes immediately under the tendons: the grooves or hollows that are thus formed on the surface of the enlargement, whilst it is in a hard state, by the friction of the tendons, evidently show that they have been impeded in their motion. In this situation, and under these circumstances, it becomes impossible to remove the ossific matter from the bone. It also frequently happens, that the legs become considerably enlarged; a case for which there is no cure, as will be evident by inspecting the bare bone, which may be found on every field or common where there are dead horses.

The same effects are also produced by those bony enlargements growing on the leg bones of horses, called splints, of which we shall speak more fully hereafter under their proper head.

Lameness also proceeds from rheumatic pains in different parts of the body, which frequently change from one part to another; also from cramps, spasms, &c.; all of which produce lameness, the true seat of which cannot be ascertained with any degree of certainty. Other causes of lameness are more easily discovered from their external appearance, or the symptoms which attend them.

Lameness also proceeds from contusions occasioned by blows; from wounds or punctures; sores upon the heels; and from what is called the scratches, salanders, and wallanders, &c. under every denomination whatever; as in all these cases the parts are more or less swelled or inflamed, and, of course, unfit for action.

Lameness will proceed also from too violent and long-continued exercise, which creates too great a waste of the synovia of the joints; hence they become stiff, and on the horse's moving, make a crackling noise.

The same effects are also produced from a variety of other causes, which are confined to the feet—as pricks from nails in shoeing, wounds in the hoofs from nails picked up in the street, glass, sharp-pointed bones, and other foreign substances, which often penetrate to the sensible parts and cause lameness.

Lameness is also often produced from contractions —from Thrushes—from Corns—from Quittors—from Sand-cracks—from paring the hoofs and sole too much, which causes all these complaints, and which will be more fully defined and treated of, under their several and proper heads.

There is also another kind of lameness peculiar to the hind legs, and which occasions a sudden jerking of the leg upwards, by many called string-halt. This complaint seems to be a particular affection of the nerves of the leg, which causes this involuntary motion, and for which it is a folly to propose a cure. I have never found it but in the best bred horses; and although people make it an eyesore in purchasing a horse, it is seldom or ever found to lame them, or impede their going. On the contrary, among the nobility of France, and those who frequent much the parade or menage, those horses are sought for, and even purchased at a very high price, but more particularly so when this affection belongs to both legs.

From all these, and a variety of other causes, lameness is produced; the true seat of which, in many cases, cannot be discovered by external appearances. Practitioners ought, therefore, to be very cautious in speaking of the causes of lameness, before they have positively discovered the real seat of the disease, or become acquainted with the circumstances which produced it. From a neglect of this precaution, together with a want of anatomical knowledge of parts, which cannot produce any thing else than a false and hasty determination as to the real seat of the lameness, applications are often made to the sound parts, and the patient's shoulder is pegged, blown and bored, by way of punishment for getting himself lame in the back sinew or coffin joint. How many pleuritic horses have been killed outright, by the cordial and spicy drenches which, probably, might have cured a colic!—and how many have been rendered incurably lame from the shoe being fixed on to the wrong foot—whilst the real cause has soon afterwards shown itself to the shame and confusion of the practitioner.

The cautions here pointed out, will no doubt appear the more necessary, when the real importance of the circumstances are properly considered, that all bony enlargements or excrescences always occasion the most severe pain to the animal whilst they are growing, which by the effect they produce in extending the membrane, or periosteum, that covers the bone, and which under certain circumstances is always more or less attended with inflammation; and I do not know of a more proper analogy, than comparing them to teething in children, which continues till the periosteum which covers the jaw bone is penetrated by the new tooth coming through.

Another cause of lameness also, which we have not yet noticed, is the injury done to horses' backs by pads and ill-made saddles, not properly fitted. I must notice also the extreme carelessness of nineteen persons out of every twenty who travel in this country, in not attending to the cloth that contains the padding or stuffing of saddles, which is generally made of coarse materials, and very often of canvas. Can any thing be more absurd?—such a covering cannot but from the friction it produces on the skin create sores and tumours of different kinds, which are very often attended with serious consequences, being extremely difficult of cure. Hence also, very large swellings are produced on the shoulder or withers, which terminate in fistulous ulcers, and which from their situation, being in want of a dependent orifice or drain, when suppuration takes place, produce evils not easily remedied; the skin becomes bruised, and hard lumps called warbles and sit-fails, render the poor animal's back tender for a long while. How often are travellers put to serious inconveniences and delays on the road, when by a trifling attention to these things they might have been avoided. How easy for a traveller, when he arrives at his journey's end, to place his saddle in the sun to dry, and before he mounts again, to have it scraped and made free from dust and dirt. However, persons who travel much on horseback, should always carry with them two saddle cloths, and these should always be made of the very best and finest serge, keeping one in his saddle bags to shift and change as often as necessary. Would not such a precaution be attended with more beneficial effects, by preventing the evil, than by travelling on till you produce one, and then be at the additional trouble of learning how to cure it?

The specific diseases which we have thus taken a pretty general view of, shall now be treated of under their several heads.

A

### COMPARATIVE VIEW

REPRESENTING THE

### DIFFERENT DISEASES

TO WHICH THE

### LEGS AND FEET OF HORSES ARE SUBJECT.

### OF BONE SPAVIN.

*Fig* 1. *letter B.*—Represents a bone spavin on the inside of the lower part of the hock, and upper end of the cuneiform bone of that part.

A BONE SPAVIN is an exostosis, or bony enlargement, entering into the formation of that part called the hock, or some part of the metatarsal bones. Its situation is not definite, but is most frequently on the upper part of the minor metacarpal (or cannon) bone. When it has the former of these situations, corresponding to splint before, it often arises from raising the outer heel by calkings; and in what are called cat, caw, or sickled-hammed horses, it is often brought on by this natural deformity; but more frequently the latter cause produces it, when on the inside of the hock. A spavin that begins on the lower part of the hock, is not so dangerous as that which puts out higher, and comes in contact with some of the small bones of the os calcis. A spavin near the edge is not so bad as that which is more inward towards the middle. From the union of the small bones, which are six in number, composing the os calcis, the elasticity is lost, and lameness must be the consequence; besides it must, as we have before observed, interfere with the ligaments, and not unfrequently with the lesser flexor tendon, producing not only an additional pain, but additional lameness.

### *Treatment and Cure of Bone Spavin.*

Various are the prescriptions laid down for the treatment of this complaint; but unless a person has attended in the dissecting room, and noticed the morbid appearance of the parts after death in this disease, he cannot be aware of the great thickening and disease the ligament takes on, as well as the bones, when bone spavin has existed for some time. It appears that this is sometimes the effect, and may probably sometimes be the consequence of the enlargement of the ligament brought on by spavin. And when the inflammation is from neglect suffered to go on, it generally communicates to the neighbouring soft parts, and by this means the periosteum, cellular membrane, ligaments and muscles, are changed into, and increase the size of the bone. In its original state, when first thrown out, it is similar unto the glutinous sub-

V. 1 p 17

stance of an egg, but in its further and more aged state it is called the adhesive ossific inflammation.

### *Cure.*

The successful treatment and cure of this disease depends much on its being attended to soon after its first appearance; for where the bony matter has been deposited, it becomes so hard and ossified, and so much a part of the organ itself, that it is difficult to stimulate the absorbents to remove it. In its incipient or first state, extensive blistering will be found sufficient, as it will stimulate both the ligament and the absorbents of the bone likewise; and if necessary must be repeated. But in its more advanced state, before it may have become bony, firing will be found necessary, and the blister well rubbed in upon it. By this means, calling all the powers to act at one and the same time, a cure may be performed.

The blister I would recommend is the following:

| | |
|---|---|
| Take Muriate of Quicksilver (corrosive sublimate) . . . . . . . | a scruple; |
| Spanish flies . . . . . . . | 4 drachms; |
| Venice turpentine . . . . . | $3\frac{1}{2}$ drachms; |
| Lard . . . . . . . . . . . | 4 ounces. |

*Mix.*

When this has been applied, and the parts nearly healed, it may be repeated, if necessary, at the end of eight or ten days, avoiding such a degree of violence or irritation as may produce a loss of hair, which in the first instance must always be cut off. But where a horse cannot have his regular treatment, being wanted for work the whole time, the following liquid blister may be applied:

Take Spanish flies (cantharides) in powder, 1 ounce; corrosive sublimate, 10 grains; Venice turpentine and sweet oil, each 2 drachms, melted together over a slow fire; add 4 ounces of the spirits. *Mix.*

Rub the part slightly, night and morning, till some running is produced, then desist. It is necessary to caution here, that although the liquid blister is recommended under the circumstances laid down, of the horse keeping at work, absolute rest or confinement in a large stall or paddock is always the best; and if you ever should apply a blister over the fire immediately after, be sure never to apply it to more than one leg at a time. The irritation produced by both being applied at the same time, might cause the death of the animal; but without the fire or actual cautery, no danger is to be apprehended.

### OF BLOOD SPAVIN.

*Fig.* 1. *letter a.*—Represents the inside of the hock, with the Varix, or Blood Spavin, and as the distended vein appears in that disease.

Blood Spavin and Bog Spavin are synonymous. Blood Spavin is a dilatation of the vein, or an encysted tumour, that runs along the inside of the hock, forming a soft swelling in the hollow parts, and is often attended with a weakness and lameness of the hock. This enlargement seldom takes place in any other part of the horse, as his superficial order is comparatively small, and not subject to such artificial pressure as our own: it is improperly considered as a disease, being only the effect of another and distinct affection. For it is affected merely from passing over the mucous capsules of the hock, which becoming enlarged, press on the vein, occasioning a passage for the circulation of the blood, and a consequent dilatation of its coats. Usually, therefore, the dilated capsule is the part to be attended to; but when the vein becomes so enlarged, as by its own pressure to occasion mischief, it can only be removed by counter-pressure, or by blister and fire.

### ENCYSTED TUMOURS,

OR

### DISEASED ENLARGEMENTS OF THE BURSÆ MUCOSÆ,

ARE

### WIND-GALLS.

*Fig.* 7. *letter a.*—Represents Wind-galls, or Encysted Tumours, that appear on the fore leg.—*a, b,* two Wind-galls that sometimes arise under each side of the knee.—*c,* a Wind-gall on the shin, between two muscular tendons, a little above the fetlock joint.

Fig. 8.—Shows Wind-galls on the hind part of the fore leg.—*a*, an uncommon one above the knee.—*b*, a Wind-gall under the knee behind.—*c*, *c*, the common and usual Wind-galls on each side the back sinew.

WIND-GALLS are a puffy kind of swellings, or tumours, which yield to the pressure of the finger, but on removing the finger recover themselves and push out as before. They have thus been named from a false notion handed down by Jack, Tom, Dick and Harry, that they contain wind or air. These tumours are often seated on both sides the back sinew of the horse, on or a little above the fetlock joint, but more frequently on the hind legs. They are quite loose and detached from the feet on which they grow, and exhibit the same signs wherever they are met with, whether in the hocks or about the knees; for these swellings are not confined to the lower limbs only, but appear on any of those parts where the cellular membrane can be separated; and although an eyesore in the purchase of a horse, they for the most part exist without occasioning pain.

Wind-galls are usually caused by riding on very hard roads, or on dry hilly grounds; sometimes by working horses too young, before their limbs have grown sufficiently firm and vigorous to support their labour. At first they are small, but sometimes grow to the size of a pullet's egg, and push out on each side of the hollow of the hock, called Thorough-pin.—Swellings of the same kind also appear on the front of the knee in the interstices of both sides of the joint, are also dangerous, and usually caused by some violent strain.

The other flatulent swellings which horses are subject to

Fig. 7.

Fig. 8.

seldom cause lameness, and are for the most part easily cured by the application of a mild blister. I mean those that happen in the interstices of the large muscles of the hip and thigh, which are generally distended like little bladders filled with air. They come by strains and over exertion, for draught horses are most subject to them.

Wind-galls that proceed from hard work or other causes, are more easily prevented than cured—by not travelling so much when too young, and with heavy riders, before they come to their full strength. On their first appearance, if the swellings ascend towards the knee, bathe them well with old verjuice or some strong astringent. This with the application of pieces of sheet lead, and a firm bandage, *put on dry*, and afterwards wetted, which adds to the pressure, will, in their incipient state, generally produce a cure. But when they are grown pretty large and feel indurate, if on the hind legs, it is hardly worth while to meddle with them.

These vascular bags, when distributed about the joints, come more into notice than others. There is a sympathy between the parts which brings them into action to supply accidental wants and deficiencies; hence it is, that increased exertions in the tendons produce increased secretions of this mucus, and this the more as by exertion a greater determination of blood is secreted; and thus it is that Wind-galls are universally the attendants of hard labour.

### *Cure of Wind-galls.*

In the cure of Wind-galls we must attend to three particulars: 1st. The removal of any diseased alteration they may have occasioned—2d. The removal of their distention, and the prevention of its recurrence—3d. Stimulating applications are the most likely to remove the coagulated deposit. The sweating blister before recommended is a very proper application for this purpose. But to promote absorption of the contents of the Wind-gall, pressure, as before recommended, generally proves the most efficacious, applied in the form of a bandage, with bolster or compress on the immediate swelling. When Wind-galls have been removed in this way, firing is the best means of preventing their return.

I cannot dismiss this subject without warning the junior practitioner, never to be incautiously led to puncture a Wind-gall, Blood Spavin, or indeed any of those bursal enlargements forming *Varix*, under the idea of evacuating its contents; for if even no mischief follow, no good can possibly result, as the cyst would be only momentarily emptied, and its capacity remain the same, by being again filled by the exhalent arteries, and inflammation of the most serious kind would be sure to follow. Horses have even been destroyed by such ignorance, and even when they are not serious they most generally end in *anchylosis;* and when by any other means such an accident should happen, through the ignorance of a smith, the owner should immediately close the wound by the heated budding iron, in the same manner as in open joints. Bursal enlargements, or Wind-galls, bear different technical names, according to their situations.

Fig. 9.

Fig. 10.

### WENS AND TUMOURS

### ABOUT THE HOCKS.

*Fig*. 9. *letter a*.—Represents a Wen, as it appears on the heel of the hock.

*Fig* 10. *a, b, c*.—Wind-galls, or flatulent tumours, that arise in all the hollow parts of the hock.

A Wen is a fleshy substance, that in different instances has been known to grow out on almost all parts of the horse's body. The causes from which they proceed are difficult to be accounted for. They begin usually in the skin, where the vessels are extremely small, and enlarging gradually, in time grow to a considerable size. They are seldom painful, being of slow growth, and are sometimes of several years standing before they grow to a large size. The substance is generally fleshy, but sometimes spongy. But when involved with fibres, they on dissection appear like so many threads laid across each other. When it has a communication with the membrana adiposa, its substance is chiefly an accumulation of a greasy matter resembling suet. One which I extracted for Mr. Benjamin Reynolds, cooper, (in Front street) from the back of a dog, nearly the size of a hat, was of this kind and substance; and I once operated for one on the eye of a horse, also one from a bullock, nearly the size of a common melon. This substance when cut was a mass of fungous flesh. There is sometimes a great effusion of blood, and it is but seldom it can be stopped by the cautery. When they become pendulous, and hang by a small root, the best way to extirpate them is by a hair, or searing it off with a hot iron, and healed with common digestive ointment, or sprinkled with a little burnt alum, and white vitriol, of equal quantities, mixed.

Young horses are ticklish, and are given to kicking when standing between bales; by these means they strike their hams and produce a swelling such as is represented in the plate upon the top of the os calcis, or hock. This, however, can be remedied by removal, and the part bathed with verjuice. I would never recommend the extirpation of these tumours when on this part.

### OF RING-BONE AND CURB.

*Fig.* 3.—Shows the hind leg of a horse in a bent position—*a*, the heel of the hock—*b*, the Curb, or hard swelling, as it appears when grown to its full size below that part.

*Fig.* 4.—Represents the fore leg of a horse in a straight position—*a*, *a*, *a*, the Ring-bone, or circular hard swelling, round the pastern joint.

*Fig.* 5.—Shows the false quarter—*a*, the seam on the quarter from the coronet to the bottom of the foot.

A Ring-bone is a bony circle surrounding the whole or part of the coronet. A Ring-bone has an affinity to a Bone Spavin, and in very upright positions, either such as are naturally so, or become so from high contracted heels, the bones

Fig. 3.

Fig. 4.

are so perpendicularly opposed to each other, that great jar is sustained during motion, and a disposition to inflammation is excited from these constant shocks.

A Ring-bone that shows itself above the coronet, producing great lameness, generally terminates in anchylosis of the joint, and renders the case hopeless. In other instances, the coronary ligament becomes ossified; in which case the lameness is not so great, though commonly very difficult of cure. But the Ring-bones that prove the least mischievous are those that are found on the sides of the foot, arising from ossification of the lateral cartilages.

In these several cases the complaint is not difficult to detect; but persons unacquainted with the anatomy of parts call them all Ring-bones without any distinction, nor can they say whether from their situation they are curable or not. The knowledge of this circumstance is of consequence to the scientific practitioner, as he not only saves the poor animal from great pain, but does not deceive his employer by the promises of a cure, when from their peculiar situation the thing is impossible.

Ring-bones are sometimes attended with heat and pain; at others with very little; but they always occasion some lameness. Those arising from ossification of the lateral cartilages are commonly confined to aged or hard-worked horses: those produced by upright pasterns, or peculiar formation of the parts, are occasioned by hard work, and generally appear at the middle period of life, while the ossification of the coronary ligament seems a constitutional affection dependent on a disposition to bony matter in these and other parts. In proof of which I have frequently seen them on colts of three and four years old.

*Treatment.*

The treatment which I have generally found the most efficacious is the following; for simple blistering will seldom succeed, and even the most judicious treatment often fails. For four or five days rub the part with the mercurial ointment; then blister; in ten days after fire, and re-blister immediately. In firing this part, you must avoid wounding the bony quick, or you may produce a Sand-crack.

### CURB.

A Curb is described among the exostoses, and is sometimes an affection of the os calcis; though more frequently an inflammatory affection of the ligament, being situated posteriorly a few inches below the point of the hock. Its existence is detected by a prominence more or less conspicuous, on the otherwise level line of the hock and cannon. It usually comes on suddenly, and generally on young horses, and is not productive of much lameness, unless very considerable, and long neglected.

*Cure.*

The cure of a Curb is seldom difficult, and if a horse can be spared only a sufficient time for the application and complete healing of a blister, he may be radically cured. They are generally the effect of a strain; and it is but to go through a regular blister, though sometimes the fire may be necessary.

### SPLINTS,

### SHOWN IN TWO VIEWS OF THE FORE LEG.

*Fig.* 5.—The hinder or back part of the fore leg, to show a Thorough Splint—*a, a,* The Splint situated behind the bone and the back sinew, appearing on both sides thereof.

*Fig.* 6.—Represents the fore leg sideways, to show the more usual and ordinary kind of splints—*a,* A Splint under the knee near the joint—*b,* A Splint on the middle and fore part of the shank bone, disfiguring the leg.—*c,* A more formidable Splint on the back part of the shank bone, near the insertion of the back sinew.

Splint is the farrier's term for a species of exostosis situated about some part of the carpal, metacarpal, or cannon bone, or in other words between the knee and pastern; very generally on the inside, but when attached to the knee itself, they call it osslet. When this bony tumour comes upon the upper part of the shank or cannon, it is universally known by the name of Splent or Splint, and which is a very common evil among young horses, but much less so among old ones. Some horses are more subject to them than others. But few put them out after six or seven years old, unless they meet with blows on those parts. A Splint that comes on the middle of the cannon or shank bone, is seldom dangerous; but those that arise on the back part of this bone, when they grow large and press on the back sinew, always cause lameness; but unless they are situated near the joint they seldom do.

### *Cure of Splints.*

As to the cure of Splints the best way is not to meddle with them, unless they are so large as to disfigure the horse, or are so situated as to endanger his going lame. Splints in their infancy should on their first appearance be well bathed with vinegar or old verjuice, which by strengthening the fibres often puts a stop to their growth; for it is the periosteum or the membrane that covers the bone that is thickened and not the bone itself; and in some constitutions purging and some diuretics will have a tendency to reduce this enlargement.

The most effectual method which I have ever seen practised is one which is in use among the nations of the East, (who understand these things pretty well) and which is followed by a celebrated veterinarian of the present day. Rub into the swelling, night and morning for five or six days, two drachms of mercurial ointment, using a good deal of friction with the hand to assist its entrance. After the mercurial friction, which leads to soften the osseous matter, apply a mercurial blister:

Take Spanish flies . . . . 4 ounces.
Corrosive sublimate . 2 drachms.
Turpentine and Lard, Q. S. to make the ointment.

And when the parts are healed, the blister may again, if ne-

29

Fig. 5.

Fig. 6.

cessary, be repeated; and in very bad cases it will be found necessary to apply the fire or actual cautery, and immediately apply the blister over it.

During my residence in India, I used to observe the natives reduce a Splint by the following mode of operation; and having made a note of this circumstance, when at the Veterinary College I tried it on several horses with great success; though many of my brother students have often laughed at me when I used to speak of it. If a Splint lies on the bone, that is, on or between the large and small metacarpal or cannon bones, so as to impede the action of the flexor tendon, which runs on the back part of the leg, I recommend by all means to let it alone; but if it only lies near that part, and on the large metacarpal or cannon bone, I would use their method in preference to any other, and a simple trial will prove its good effects. First rub the part with a round hard stick or buckhorn handle of a knife, until you feel it somewhat softer than before, then take a large darning needle, and fix it in a small hand-vice so as to make it manageable; heat it over a candle, and when moderately warm, make according to the size of the Splint, several (say three, four, or half a dozen) holes, being sure that some of them reach the bottom; then apply a gentle blister, and repeat it, if necessary.

I have never tried it on a Bone Spavin, but I should think, from the similarity of the two, that in the very early stage of the complaint, before ossification has really taken place, that it would have the same effect. It is at all events worthy of trial.

### QUITTOR,

### OR FALSE QUARTER.

*Fig.* 11.—Represents the Quittor—*a*, the orifice from whence the matter generally flows—*b*, the matter running down the quarter—*c*, *c*, the swelling round the coronet—*d*, the sinking and depression of the hoof, caused by the erosion of the ulcer.

Quittor, or Quittor-bone, is by Mr. Coleman defined as an ulcer between hair and hoof. It arises from treads and bruises on the inside of the horse's hoof; sometimes from gravel, which, by working its way upwards, lodges above the coronet; in this country called Gravelling.

Mr. Youatt, a celebrated practitioner of the day, taught me while at College a great deal respecting this dreadful and painful complaint.

It is not an uncommon disease, being a very destructive one which has always occupied a considerable place in the farrier's list. The older ones in all countries, impressed with the obstinacy of its character, had always employed violent means for its cure. Monsieur La Fosse, in the early day of his practice, many years ago, thought it a complaint of the cartilages. But this idea was found to be erroneous; for cartilages are vascular, and as such they must be capable of living action, though it is slow; and hence, where disease exists, they will exfoliate like other parts.

The practice of La Fosse was received into England with avidity, and progressed until the first professor of the Veterinary College, Mr. St. Bell, came to England, when another treatment was introduced.

It is now well known that parts of little vascularity, when diseased, require stimulating, and sometimes very actively; for it is necessary first to destroy the diseased surfaces, and then to excite the healthy action, to enable them to throw off the destroyed portions. Formerly, as we have noticed, either the actual cautery or caustic were employed for this purpose, though the practitioners were not aware of the rationale of their operations. Of late date the knife has been used, and by bringing the part into a simple wound, promoting a natural cure. Its very premises, Mr. Youatt says, are wrong; the simple exciting of organs so little vascular as those in confirmed Quittor, will not bring the parts into a simple wound, from the difficulty of removing the whole of the sinuses with the knife; for it is well known that if any of these are left unexercised, the disease is not subdued. It is further ineligible, from the great danger of wounding the capsular ligament in making the necessary sections, particularly when the sinuses run inwards.

I have before stated that a wound in the cavity of the foot only becomes a Quittor when it has taken on a peculiar diseased ulcerative process, principally dependent on the parts it affects.

Quittor is one of the most difficult diseases of the foot we have to contend with. It takes its origin from several causes; such as pricks, an overreach; but with draught horses it most generally is occasioned by a bruise or a wound inflicted on the horse by a tread on the coronet; and which circumstance happens more frequently in this country than any other, in sleighing time, when horses are always shod with calking—an erroneous idea, that horses cannot go in winter time without them. *There is, however, a method of shoeing horses to prevent all these evils; but this is a secret in the shoeing art which I have never yet unfolded:* for I do positively assert that a horse may be shod during the winter, and be made to go as safe for twenty years without ever causing a Quittor, as if he had a calking on each shoe. But when you tell a blacksmith in Philadelphia how this is to be done, or attempt to introduce any thing new, which they have never seen before, they immediately cry out, "O Doctor, I knew this before; I shod horses in this way, 20 years ago, in Ireland and Scotland!"—This, however, is foreign to our subject.

The high calkings of their shoes are peculiarly mischievous, and often create serious evils. Any part of the upper margin of the foot is open to this accident, but one of the quarters is most usually affected; and as the injury is mostly considerable, so the lateral cartilages are at once wounded, and if not, the bruise being such as to produce death in a portion of the surrounding integuments and other vascular parts, the coronary expansion, &c. extensive ulceration soon follows, and the dead portion being thrown off by the inflammation, farmers and others ignorant of those things say a core has come out. At this period of the complaint it is however evident, that the disease should be treated in no respect as a confirmed Quittor, but simply wound or abscess; and it is to a different consideration of the subject, that the future evils result, for farriers are too apt on those occasions, under the idea of assisting the core out, to introduce strong stimulants, the inflammation excited by which actually occasions the evil they intend to prevent, for the less vascular parts then take on the disease, and sinuses begin to form. If, therefore, matter begins to form, and penetrates in a direct line downwards, make an opening in the hoof below, and place the whole foot in a cooling poultice; avoid motion of every kind, and use other means of combating the inflammation, as bleeding, low diet, and internal remedies.

### *Treatment of Confirmed Quittor.*

When this complaint has fully established its character, then it can no longer be considered as a bruise, wound, or abscess, but must be regarded as an ulcer, which must be cured by means more consistent with the modern forms of surgery. But after all, no method has been found so successful as the long established practice of coreing it out, which is nothing more than the caustic process to destroy the ulcerated edges, and by raising a strong inflammation produce a new and healthy action.

The first necessary step to the cure, is a careful examination, with a *leaden probe*, of the extent of the ulcer or pipes; and should any of these proceed inwards, and the probe meet with a hard body, it will be more than probable the bone is bent, and the case will turn out unfavourable. But when, on the contrary, the sinus is outward and downward, or backward towards the heel, the cure will not be so difficult.

From what has now been said on this difficult subject, it will be evident that the substances that act as a caustic, may be introduced to remove the diseased surface, or according to the farrier's term, to core out the Quittor. Crystallized verdigris, corrosive sublimate, butter of antimony, arsenic, solutions of potash, and lunar caustic, are all in use for this purpose. However, one circumstance is self-evident, which is, that humanity as well as prudence dictates that we should use the mildest means first. It too often fails; but in every instance where the sinus can be reached by a syringe, the cure should be attempted by an injection for a few days with a tincture of cantharides in turpentine, or a mild solution of caustic alkali or lunar caustic; but if these should fail, it will be prudent to proceed to the more active stimulants. When the upper openings are very small and the sinus deep, make a paste with equal parts of rosin and sublimate, softened with tar; impregnate small pieces of wool fully with this paste, which place round the end of the probe, and introduce them, but not jam them in with force. Sublimate and verdigris rolled up in thin paper is frequently used at the college so as to make a bougie, which being greased with tar to make it slippery, should be introduced to the bottom of the sinus. The hoof also should be thinned and put into a poultice, by which the diseased surface may be dressed. In two or three days after the introduction of the caustic, great heat and tumefaction of the foot will follow, and at last it will burst open, expelling

35

the slough, together with the application which occasioned it, after which process the wound will heal.

### FALSE QUARTER,

### OR SAND-CRACK.

This consists of a separation of the horny fibres of the hoof, in a perpendicular direction; and if they extend to the coronet they are extremely difficult to cure. The term Sand-crack is supposed to arise from sand and dust getting into the fissure or opening of the hoof, and which is erroneously supposed to be the cause.

The old nomenclature of Farriery has many absurd names, but this has a more significant origin, and is called Sand-crack because it is ever supposed to be peculiar to hot sandy districts. This however is erroneous, for no horses are less liable to Sand-cracks than the Arabs, before they get into our possession, and become subjected to our stable management of standing on litter; and if people would only get into the habit of using sand more frequently than straw, Sand-crack would soon disappear—but here again the servants would interfere and crush it as an absurdity. These fissures are more common to the fore than the hind feet, but in cart horses they most generally come in front of the hoof, which I say is no Sand-crack, but a fissure in front. Sand-cracks, as they are called, are caused by contraction, which originates from a brittleness of the hoof for want of the proper application of water to the feet, and against which the new veterinary bath will prove a sure preventive; for since the introduction of water by the bath into the cavalry regiments in Europe, no such thing as Sand-crack is now known or even heard of. Sand-crack, or what I more properly call a fissure of the quarter arising from contraction, originates from an inflection of the quarter of the hoof round the points of the coffin bone, and which is sufficiently explained in my treatise lately published, on Contraction.

The fissure is not always of one determinate depth, being sometimes so superficial as not to penetrate the whole thickness of the horn, and occasions no inconvenience at first. At others it extends through the horn, and comes to the sensible laminæ underneath. Neglect and a continuance of work, however, soon produces mischief, and aggravates by the introduction of dirt, sand, or gravel; it then becomes a painful affection and produces great lameness, and from the injury done to the sensible laminated expansion, there is often sprouting fungus between the divided edges which greatly aggravates the complaint. Accidents of all kinds, injure the vascular origin of the hoof. They also sometimes originate from stubs, treads, &c.

### *Treatment of Sand-cracks.*

The grand object is to interrupt the communication between the crack and sound hock, and a radical removal of the evil will be best done by scraping the divided edges with a drawing knife, thinning the quarter with the rasp, and the application of the bath provided the opening does not come in contact with the laminæ; if it should, the bath must not be used until the laminæ is quite protected by a renewal of the horn.

When a Sand-crack is the effect of an injury done to the coronet, the rising edges of the horn must be reduced, and the surrounding portion also thinned. The college treatment is then to fire transversely of sufficient depth so as not to touch the laminæ, which will stop the extension of the line or fissure. If, however, dirt or sand has penetrated into the opening, it must be carefully removed, and the wound dressed with tincture of myrrh and aloes: then close or bind it up until sufficiently healed to leave the bandage off. If suppuration has made its way and penetrated the horn, introduce a small quantity of the lunar caustic in solution, as ten grains to a drachm of water; bandage the hoof up for two or three days, then again examine the fissure; if then the oozing is stopped, and no inflammation appears, proceed to draw a transverse line of moderate depth across with a firing iron above or below. I have, however, frequently performed this with the rasp or drawing knife; but the iron is best, the seared line being more strong than the rasped, or the one cut with a knife. The horse must now be shod with the *patent bar shoe*, as represented in the plate of my other work on contraction; so that the hoof underneath may not touch the shoe. The quarter being now rasped, and the frog made to press on the most prominent part of the middle of the heel of the shoe, will cause the quarter to expand, and the fissure will thereby close.

If the fissure should now be sufficiently closed to warrant your turning him out, do so; but if he is kept in the stable, he must have walking exercise daily *in hand.* It now only remains to be noticed here, that as most of these cases take their origin from an altered condition of the hoof, for want of the proper and timely application of the bath, when young and before any contraction has made its appearance, so all preventive means detailed under contraction apply here till after the recovery is completed.

### OF CANKER.

Canker is a diseased, debilitated, and vitiated action of the blood vessels, that in a state of health are meant for the secretions of the sensible sole of the foot.

From the very great vascularity of the internal part of this organ, and likewise from the nature of its office, many of them being secretory organs, the inflammation that occurs in them prove, in progress and effect, altogether different from those that take place in other parts. The great vascularity heightens the violence of the inflammatory actions, and from their secretory structure it happens that when they become inflamed a great increase of part arises, which is never of a healthy nature, but commonly compounded. Canker is a very obstinate and destructive disease, as much if not more so than any of the diseases that the veterinarian has in the whole course of his practice to contend with; and Mr. Coleman as well as Mr. A. Cooper say that they know of no disease of the horse that comes more in affinity with the human subject than that called Cancer in the breasts of women: and the Greek and Roman writers of ancient days knew well what this dreadful disease was, and they called it by various names, particularly that of *lupus*, because it eats away the flesh like a wolf. This is completely realised in the disease called Canker in the foot of a horse.

This disease may be brought on in various ways. A very common origin is from a neglected Thrush, in which the suppuration, extending beyond the sensible frog, inflames the vascular sole, and extensive ulceration succeeds. Virulent and neglected Grease will sometimes occasion it. In both these ways it is frequently engendered among heavy cart horses, particularly where many stand together in crowded and confined situations, as those of coal porters, brewers, &c. Here they become greased and thrushy, from wet and neglect, and Canker soon follows. It also often arises from pricks; and where such an injury extends to the flexor tendon and Canker is the consequence, it is commonly of the worst kind; though more frequently the poor animal, under such circumstances, is cut off by the Lock-Jaw, or by the Hectic, arising from the extensive sloughing. Treads, bruises or bad corns may now and then occasion it. It is but seldom met with in the fore feet, and but seldom or ever met with in this country, though it is perhaps a more common disease than any we have in England and on the continent; at the same time Canker and Thrushes are very frequent in this country, without ever extending to the sole: but from what cause this is occasioned I am unable to account for.

### *Treatment and Cure of Canker.*

Mr. St. Bell, the first professor of the Veterinary College, pointedly insisted on the total removal of the insensible sole and frog; but as the pain necessarily attendant on such a cruel and barbarous operation, from the very firm adhesion of the parts, and as experience has proved that a more mild treatment will suffice, it is of course not required to go to such extreme severities. And when Canker is only in its infancy, every diseased portion of it may easily be removed by a drawing knife or scalpel. The surface of the diseased sole and frog must then be accurately examined, and every part of the fungus production removed, which sometimes will produce considerable hæmorrhage, and which may be checked by slightly touching it with butter of antimony, or the actual cautery; then carefully examine what extent of horny sole is separated from the sensible sole, or as a farrier would say, how much it is underrun; exactly to this extent must the sole of the hoof be removed with the drawing knife; for it must never be lost sight of, *that the horny sole once separated never reunites*, but becomes a foreign body, and as such, occasions the same effects that occur from the presence of foreign bodies in all other parts, namely, irritation, and an inflammatory process to attempt the removal of foreign substances. Every portion, therefore, of separated horn must be carefully removed: not only at the first, but at every *future* dressing, for even the smallest particle left, although only as big as a pin's head, will again extend itself all over the sole.

Having thus fulfilled the first indication by removing the diseased fungus, and having lessened the irritation which occasioned it, by removing the detached horn, the next process is to promote a more healthy action in the diseased surface.—Two plans tend to this end; the first is, by stimulants applied to the surface of the vessels; the second is by pressure, which strengthens them greatly and generally. But as long as there is a profuse secretion of whitish matter, and as long as the fungus sprouts beyond the surrounding parts, so long will the cankerous action be going on, and during this time no secretion of sound horn will ever take place, an unhealthy formation of thin half formed horn may be observed over many portions of the surface; but this will always prove an *unhealthy secretion*, and must not be allowed to remain, but on the contrary be carefully removed at every dressing, until by the application of caustic stimulants and the benefit of pressure, a granulating surface is formed which produces only good pus or matter, and finally ends in the formation of sound horn.

After the exposure of the whole cankered surface and the treatment before directed, let it be sprinkled with either of the following powders:

No. I.

| | | |
|---|---|---|
| Red precipitate . . . . . | half an ounce | |
| Verdigris . . . . . . . . | Do. do. | |
| Calomel . . . . . . . . . | Do. do. | *Mix.* |

No. II.

| | | |
|---|---|---|
| Blue vitriol . . . . . . . | one ounce | |
| Burnt alum . . . . . . . | Do. do. | |
| White lead . . . . . . . | Do. do. | *Mix.* |

The affected parts being covered with either of these stimulants judged proper, let it be dressed as dry as possible by first laying lint on the immediate surface, and then pledgets of tow thickly over the bottom of the foot, which should be done very judiciously, so as to keep up even pressure on the whole; and in order to do this the more effectually, Mr. Coleman and Mr. Youatt would direct strips of iron or steel slid under the shoe crosswise, which will not only retain the dressing, but promote pressure. This being done, wrap up the whole foot in a sacking of thick cloth or a boot. It is a very great fault in dressing cankered feet but too seldom: no case ought to be dressed less than every day; and very bad cases twice a day. From a wish to avoid trouble this is neglected, and the cure rendered incomplete. Keeping, therefore, the before above mentioned indication, the practitioner at each future dressing cannot be at a loss how to proceed, and when the fungus is completely reduced and the discharge lessened in quantity, and it has become healthy in appearance and quality, with a sprouting healthy horn, then nothing more is necessary than to wash the parts, *to dress dry*, and give a proper degree of pressure till the sore is completely healed over.

A fine chesnut horse, formerly the property of general Boyd, had this disease so very bad during my residence on Long Island, that it took nine months before the animal was completely cured. This was an extraordinary case, and excited the attention and surprise of many hundred gentlemen in New York.

## Figures and Tables

**Fig. 1. f1:**
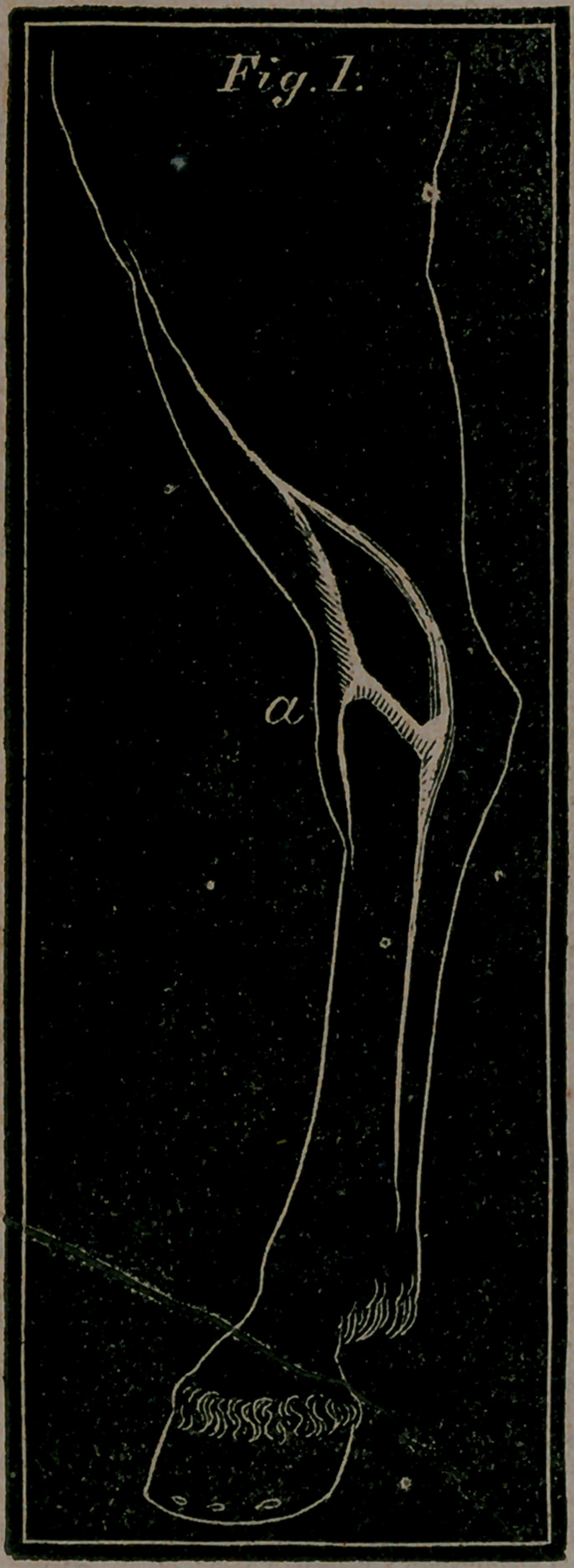


**Fig. 2. f2:**
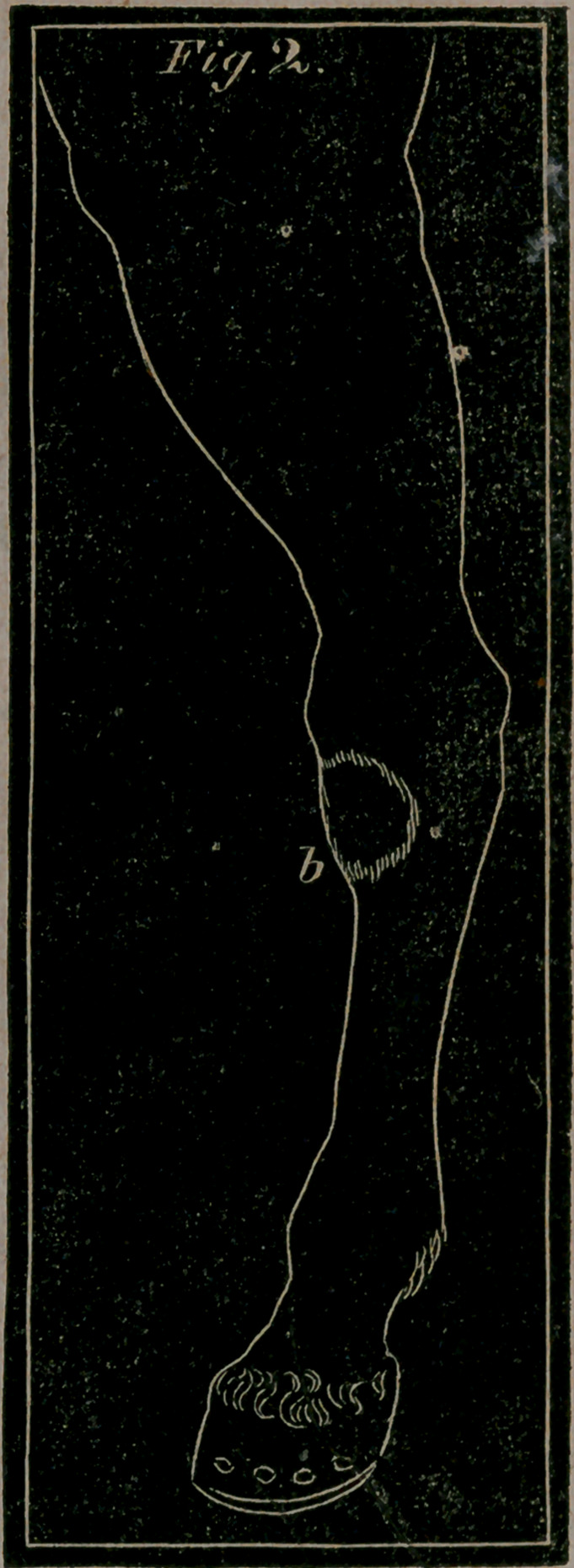


**Fig. 7. f3:**
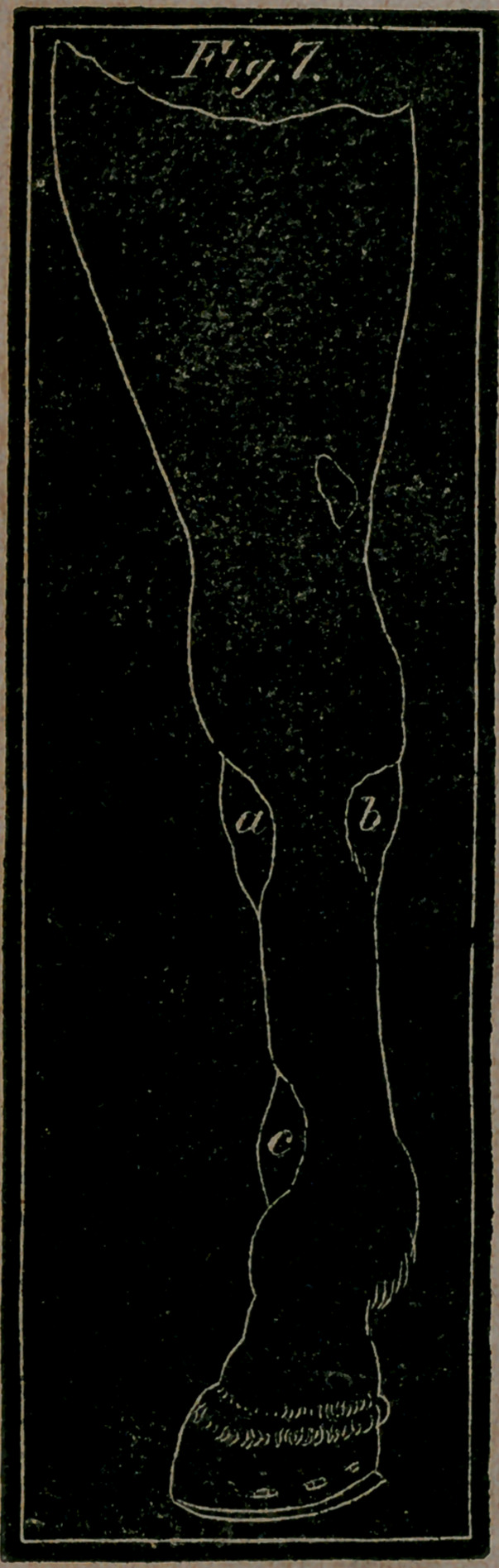


**Fig. 8. f4:**
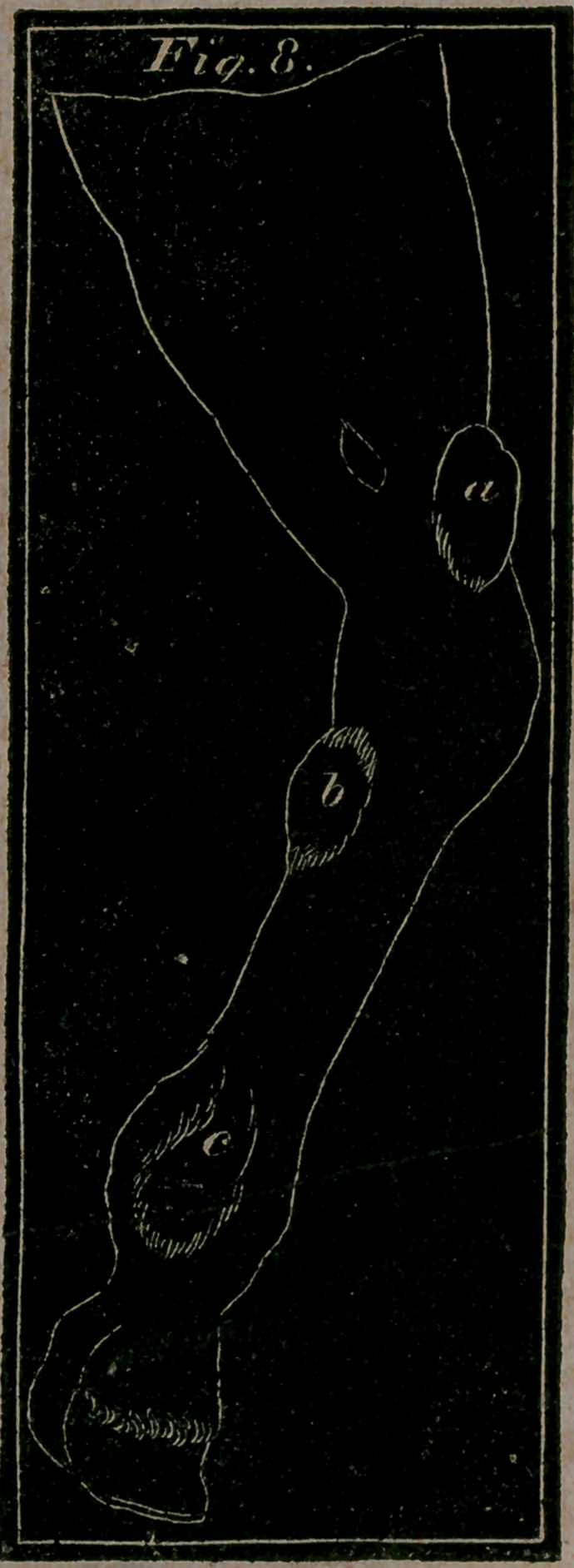


**Fig. 9. f5:**
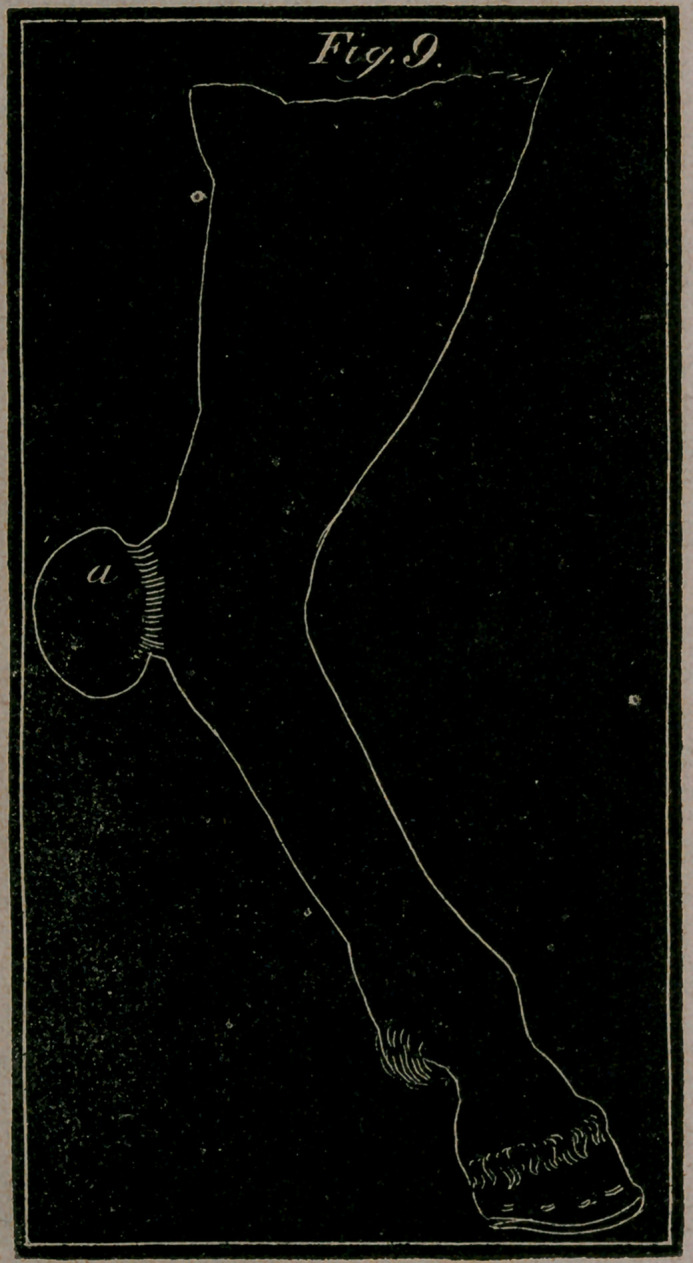


**Fig. 10. f6:**
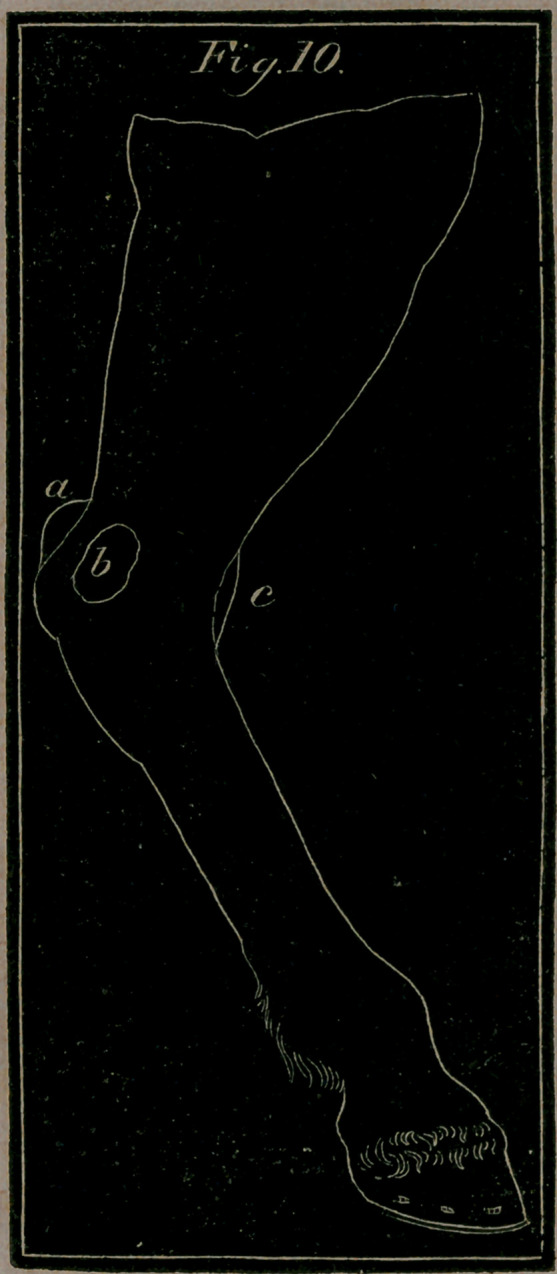


**Fig. 3. f7:**
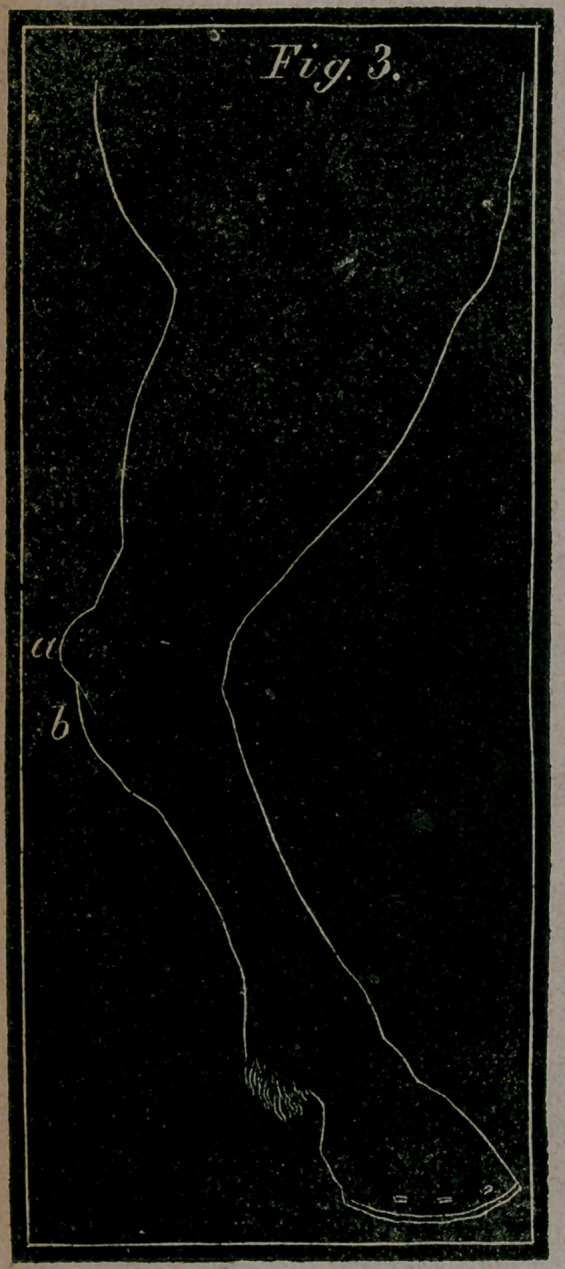


**Fig. 4. f8:**
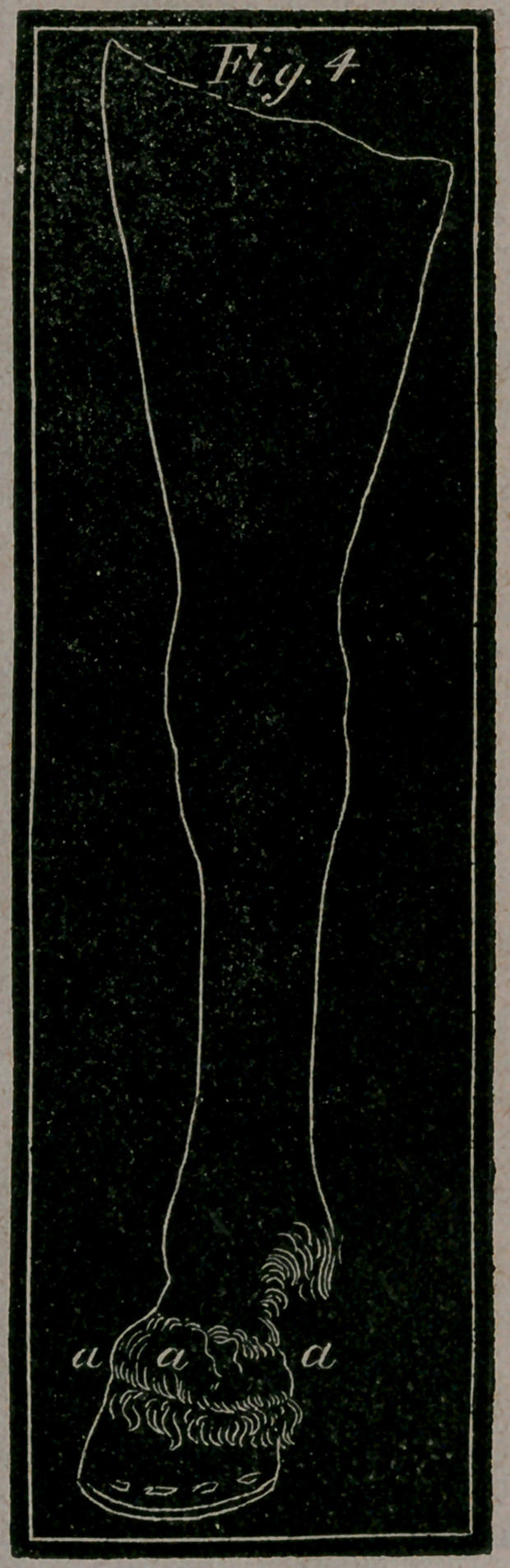


**Fig. 5. f9:**
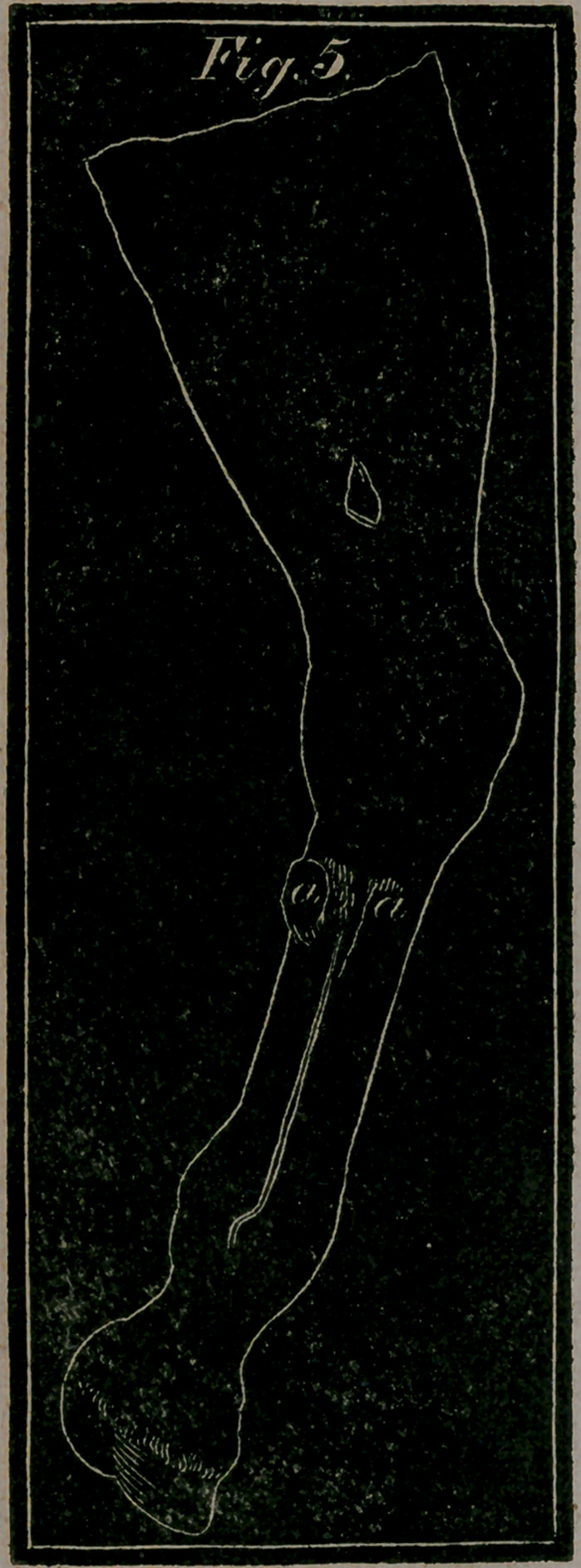


**Fig. 6. f10:**
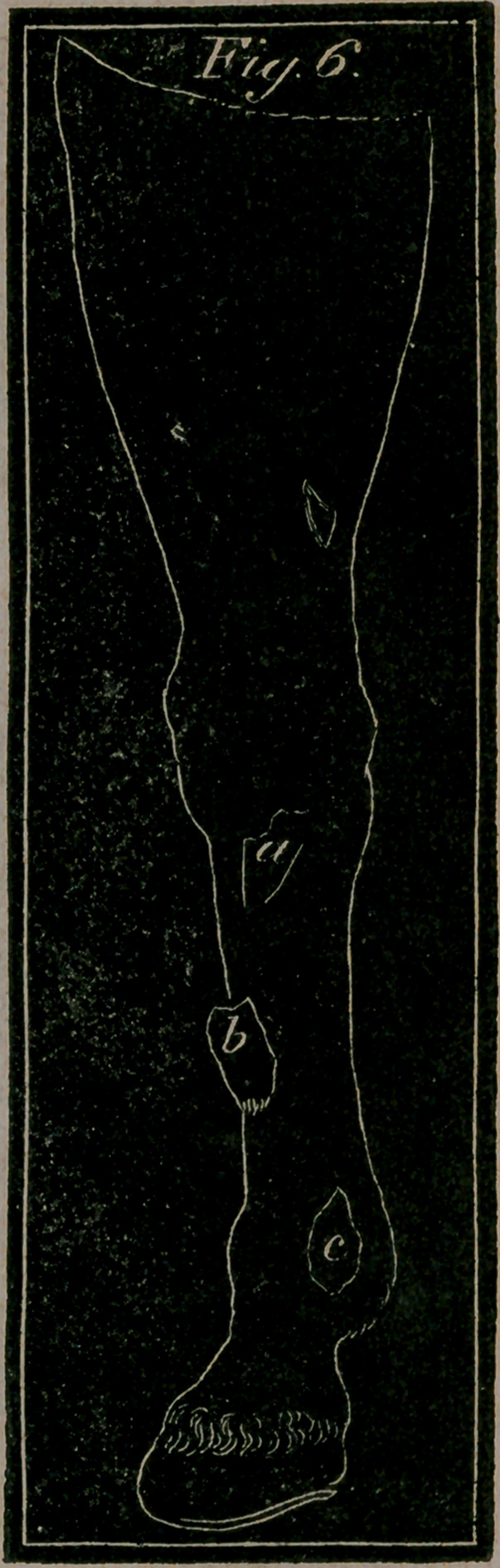


**Fig. 11. f11:**
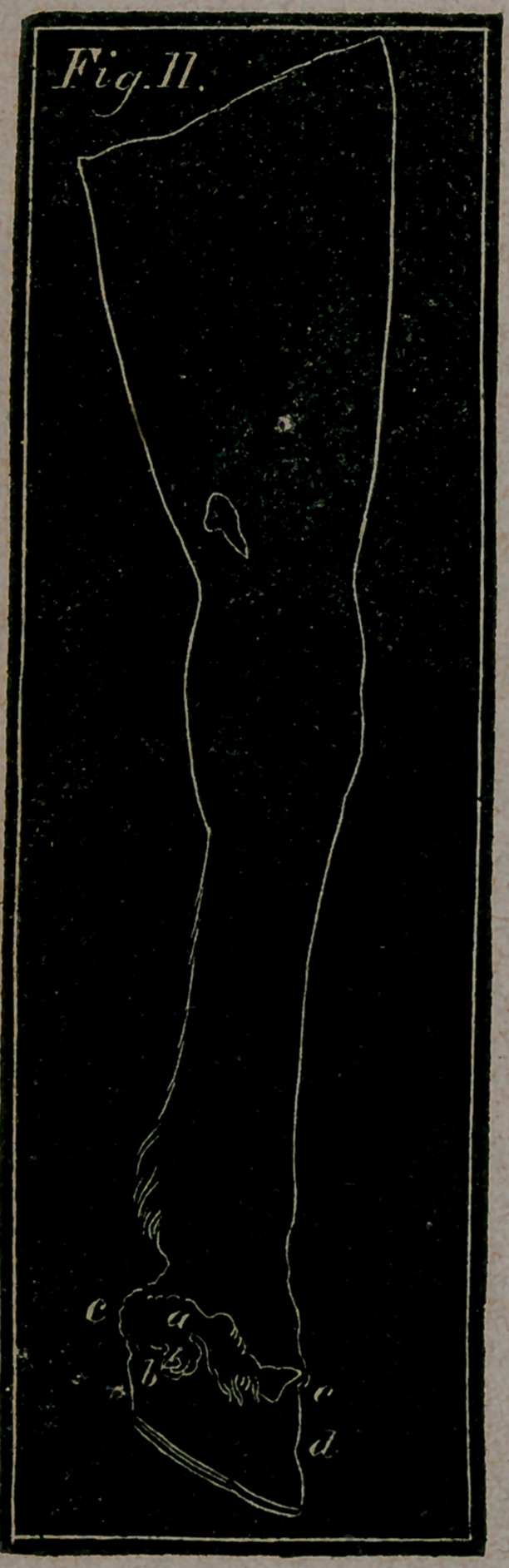


**Fig. 12. f12:**