# Errata

**Published:** 1818

**Authors:** 


### *ERRATA.*

Page 15—For *Fig.* 1. *letter B.* read *Fig.* 2. *letter b.*

In Plates.—fig. 5. letter *a* omitted on the false quarter.
fig. 6. letter *c* represents a Windgall instead of a Splint.
fig. 12. represents a Sand-crack at letter *a*.

